# Heat Stress Triggers Differential Protein Accumulation in the Extracellular Matrix of Sorghum Cell Suspension Cultures

**DOI:** 10.3390/proteomes8040029

**Published:** 2020-10-22

**Authors:** Mamosa G. Ngcala, Tatenda Goche, Adrian P. Brown, Stephen Chivasa, Rudo Ngara

**Affiliations:** 1 Department of Plant Sciences, Qwaqwa campus, University of the Free State, Phuthadithjaba 9866, South Africa; ngcalamg@gmail.com; 2 Department of Crop Sciences, Epoch Mine Campus, Gwanda State University, Filabusi, Zimbabwe; tatendagoche@gmail.com; 3 Department of Biosciences, Durham University, South Road, Durham DH1 3LE, UK; a.p.brown@durham.ac.uk (A.P.B.); stephen.chivasa@durham.ac.uk (S.C.)

**Keywords:** sorghum, cell suspension cultures, heat stress, heat shock proteins, secreted proteins, quantitative proteomics, gene expression analysis

## Abstract

Plants reprogram gene expression as an adaptive response to survive high temperatures. While the identity and functions of intracellular heat stress-responsive proteins have been extensively studied, the heat response of proteins secreted to the extracellular matrix is unknown. Here, we used *Sorghum bicolor*, a species adapted for growth in hot climates, to investigate the extracellular heat-induced responses. When exposed to 40 °C for 72 h, heat-sensitive Arabidopsis cell suspension cultures died, while ICSB338 sorghum cell cultures survived by activation of a transcriptional response characterized by the induction of *HSP70* and *HSP90* genes. Quantitative proteomic analysis of proteins recovered from cell culture medium revealed specific heat stress-induced protein accumulation within the sorghum secretome. Of the 265 secreted proteins identified, 31 responded to heat (≥2-fold change), with 84% possessing a predicted signal peptide for targeting to the classical secretory pathway. The differentially accumulated proteins have putative functions in metabolism, detoxification, and protein modifications. A germin (SORBI_3003G427700) was highly heat-inducible at both protein and gene level. Overall, our study reveals new insights into sorghum responses to heat and provides a useful resource of extracellular proteins that could serve as targets for developing thermotolerant crops. Data are available via ProteomeXchange with identifier PXD021536.

## 1. Introduction

Temperature is an important external factor controlling growth and geographical distribution of plants [1]. Different plant species tolerate specific temperature ranges for normal growth and physiological functioning [2,3,4,5]. Outside these optimal ranges, plants experience varying levels of temperature stress [5], depending on the duration of exposure, intensity, and developmental stage [2,6]. In agroecosystems, high temperatures negatively affect plant growth and yield [7]. Future losses in agricultural productivity are inevitable as climate change is predicted to increase both ambient temperatures and frequency of drought spells [8]. Therefore, it is important to study plant heat stress responses for the development of improved thermotolerant crops.

High temperature or heat stress is defined as an increase in temperature above the optimal threshold [9,10]. It inflicts damage in various physiological/molecular processes and plant organs, including leaf burn, senescence and abscission, inhibition of root and shoot growth, fruit damage, protein denaturation, and changes in membrane integrity [6,10,11]. In extreme cases, heat stress may result in cell injury and plant death [5,12]. In order to survive periods of heat stress, plants utilize specialized mechanisms to protect cellular machinery from heat damage, maintain cellular homeostasis and promote thermotolerance [12,13]. For example, under heat stress, plants alter their gene expression [14], reducing the synthesis of “normal” proteins, while promoting that of heat shock proteins (HSPs) and other stress-related proteins [12,15,16]. Excellent reviews on plant heat stress responses are available for further reading [3,9,10,17,18].

HSPs, including HSP100, HSP90, HSP70, HSP60, and small HSPs, are a group of proteins induced by diverse stress factors, including heat [16,19]. They function as molecular chaperones, as they are involved in protein folding, assembly, translocation, and stability [16]. In sorghum seedlings, an increase in growth temperatures from 35 °C to 45 °C resulted in suppressed synthesis of “normal” proteins coupled with increased accumulation of HSPs [15], possibly for thermoprotection of cellular structures. Other heat-induced stress-proteins and molecules include antioxidant enzymes and secondary metabolites, which detoxify reactive oxygen species [13,18], as well as compatible osmolytes for osmoregulatory functions [18,20]. Apart from the above-mentioned cellular changes, heat sensing and signal transduction pathways play critical roles in perceiving fluctuations in environmental temperatures and in regulating transcriptional changes for specific responses to be elicited [21,22,23]. However, the precise identities of cellular molecules and/or structures that sense heat stress together with their modes of action are yet to be fully understood.

Recently, Janni et al. [24] comprehensively reviewed heat stress transcriptomic, proteomic, and metabolomic studies of a variety of agriculturally important crops. Among the cereals, maize (*Zea mays*), rice (*Oryza sativa*), and wheat (*Triticum aestivum*) have received considerable attention in the proteomic analyses of total soluble protein of various tissues and organs in response to heat [24], with none being reported on sorghum (*Sorghum bicolor)*. Sorghum itself is naturally well-adapted to hot and dry environments [25,26] where many other food crops fail to produce maximal yield. Sorghum varieties also display diverse phenotypic traits [27,28], which are useful sources of variation in comparative stress response studies [29]. Despite the available genome sequence [30], the full genetic potential of sorghum as a model system for tropical grasses [31] is yet to be exhaustively explored.

In the current study, we focused on the effects of heat stress on the differential accumulation of secreted proteins of a sorghum cell suspension culture system using non-gel based proteomic tools. Secreted proteins are located outside the cell, in the extracellular matrix [32] where they are involved in various processes including signaling, detoxification, and defense, as well as general response to biotic and abiotic stresses [33,34,35]. Our working hypothesis is that, when plants are exposed to stress, secreted proteins are recruited for signaling and protective functions, hence our interest in identifying differentially accumulated proteins during adaptation to heat stress.

## 2. Materials and Methods

### 2.1. Plant Material and Heat-Stress Treatment

ICSB338 sorghum (*Sorghum bicolor*) cell suspension cultures initiated from shoot derived, dark-grown callus [36] were used in this study. Although the heat phenotypic trait of ICSB338 is currently unknown, this sorghum variety is relatively more susceptible to salt [37] and drought [38] stresses compared to other sorghum varieties. Cell cultures were grown and maintained under continuous darkness with agitation at 27 °C (±2 °C) every 10–12 days and sub-cultured for at least three generations prior to stress treatments. Eight-day old ICSB338 sorghum cell suspension culture, growing at mid-log phase [36] were used for the heat stress experiments. Triplicate 50 mL cell culture samples each for the control and heat stress treatments were generated. Control cell suspension cultures were kept at 27 °C, while heat-stressed cultures were incubated at 40 °C for 72 h. During the 72-h incubation period, cell suspension cultures were sampled for the determination of cell viability and extraction of total RNA and secreted proteins. For cell viability and gene expression analyses, samples were harvested in a time-course experiment at 0, 24, 48, and 72 h, and at 72 h for protein extraction.

Arabidopsis (*Arabidopsis thaliana* var Erecta) cell suspension cultures available in Dr. Stephen Chivasa’s laboratory, Durham University, UK, were maintained under continuous illumination at 22 °C as described previously [39]. The cell cultures were subcultured every 7 days, and 3-day old cultures, growing at mid-log phase, were exposed to heat stress at 40 °C for 72 h, while control samples were kept at 22 °C. In the present study, Arabidopsis cell suspension cultures were only used for the estimation of cell viability at 0, 24, 48, and 72 h following the heat stress treatment.

### 2.2. Cell Viability Estimations Using the MTT Assay

The viability of heat-stressed sorghum and Arabidopsis cell suspension cultures was estimated using the 3-(4,5-dimethylthiazolyl2)-2,5-diphenyltetrazolium bromide (MTT) assay as described previously [40]. Three biological replicate 8-day old sorghum and 3-day old Arabidopsis cell suspension cultures were prepared for the control and heat stress treatments. For each of the biological replicates, two technical replicates of 150 μL cell cultures were sampled at 0, 24, 48, and 72 h for cell viability estimations.

### 2.3. Protein Extraction, iTRAQ Labelling and Cleaning-Up

Secreted protein extracts enriched in the culture filtrate of sorghum cell suspension cultures were used for isobaric tags for relative and absolute quantitation (iTRAQ) analysis. Control and heat-stressed cell cultures were harvested after 72 h of heat stress treatment and filtered through four layers of Miracloth. Secreted proteins were extracted from the culture medium using acetone precipitation and centrifugation techniques, followed by solubilisation in 9 M urea, 2 M thiourea and 4% (*w/v*) CHAPS as described previously [41,42]. Four biological replicate samples each were prepared for the control and heat stress treatment.

The protein samples were digested with trypsin and the peptides labelled with iTRAQ tags as described previously [42] with minor modifications. Briefly, 12.5 μg of protein from each sample were acetone precipitated and processed using the iTRAQ Reagent-Multiplex Buffer Kit (AB Sciex, Redwood City, CA, USA) according to the manufacturer’s instructions, with minor modifications. The protein samples were resuspended in the sample buffer, reduced, alkylated, and digested overnight at 37 °C using 1:10 (*w/w*) trypsin (Promega Corporation, Madison, WI, USA) to protein sample ratio as described previously [38]. The digested samples were vacuum-dried, re-suspended in triethylammoium bicarbonate buffer (pH 8.5), and labelled with an 8-plex iTRAQ reagent kit (AB Sciex) according to the manufacturer’s instructions. Peptides of the four control samples were labelled with isobaric tags 113, 114, 115, and 116, while the heat-treated samples were labelled with tags 117, 118, 119, and 121.

The eight isobaric-tagged control and heat-stressed samples were pooled into one composite sample, vacuum-dried and cleaned-up using the Hydrophilic Interaction Liquid Chromatography (HILIC) SPE cartridges (PolyLC Inc., Columbia, MD, USA) as described previously [29]. Bound peptides were eluted from the cartridges, freeze-dried, and re-suspended in 3% acetonitrile (ACN) and 0.1% formic acid (FA) for liquid chromatography-tandem mass spectrometry (LC-MS/MS).

### 2.4. LC-MS/MS and Mass Spectra Data Analyses

LC-MS/MS and mass spectra data analyses were performed using previously described detailed protocols [29], with minor modifications. LC-MS/MS was conducted on peptides originating from 5 µg protein, using a Triple TOF 6600 mass spectrometer (AB Sciex, Redwood City, CA, USA) linked to an Eksigent 425 LC system via a Sciex Nanospray III source (AB Sciex, Redwood City, CA, USA). Spectrometer data was acquired using the Analyst TF 1.7.1 instrument control and data processing software (AB Sciex, Redwood City, CA, USA).

For protein identification and relative quantification, the raw.wiff data-files were processed against the UniProt protein sequences of *Sorghum bicolor* only (downloaded in May 2018) using ProteinPilot 5.0.1 version 4895 software (AB Sciex, Redwood City, CA, USA), incorporating the Paragon Algorithm 5.0.1.0.4874, (AB Sciex, Redwood City, CA, USA). All proteins identified on the basis of a single peptide were filtered out of the dataset, resulting in a total of 265 ICSB338 sorghum secreted proteins. For quantitative analysis of the heat stress responsive proteins, the abundance of each protein in all samples was calculated as a ratio of the 113-tagged control sample. Thereafter, an average ratio of each protein was calculated across all four biological replicates. For the down-regulated proteins, the average fold change of the heat-stressed was the numerator, while that of the control was the denominator. All down-regulated proteins were denoted with a negative sign. A Student’s *t*-test at *p* ≤ 0.05 was used to calculate the probability values of the differentially accumulated proteins and a cutoff threshold of two-fold was applied to filter the dataset. We further applied the Benjamini–Hochberg procedure with a stringent 1% significance level on the 265 identified proteins in order to control the false discovery rate in multiple comparison testing [43,44] The mass spectrometry proteomics data have been deposited to the ProteomeXchange Consortium via the PRIDE [45] partner repository with the dataset identifier PXD021536.

### 2.5. Bioinformatic Analyses

The presence/absence of signal peptides in protein sequences of the heat stress responsive proteins and the Biological process Gene Ontology (GO) terms were determined using data available on the UniProt database [46], while family names and conserved domains were determined using data available on the InterPro database [47].

### 2.6. Gene Expression Analysis

Total RNA was extracted from control and heat-stressed ICSB338 sorghum cell suspension cultures using the Spectrum^TM^ Plant Total RNA Kit (Sigma Aldrich, St. Louis, MO, USA) according to manufacturer’s instructions. Complementary DNA (cDNA) synthesis was performed on 1 μg total RNA template using the GoScript™ Reverse Transcriptase System (Promega, Southampton, UK) according to the manufacturer’s instructions. The qRT-PCR reaction mixtures were prepared using a 20-fold dilution cDNA and the SensiFAST™ SYBR No-ROX Kit (Bioline, Nottingham, UK) as described previously [38]. The qRT-PCR reactions were run on a Corbett Rotor-Gene 6000 (Qiagen, Cambridge, UK) using the previously described thermal cycling conditions [29]. All reactions were carried out for four biological replicates, each with three technical replicates. Data analysis was performed using the REST2009 software version 2.0.13 (Qiagen) with two sorghum genes, an EIF4a1 Sb04g003390 [48] and an uncharacterized Sb03g038910 [49] used as constitutive reference controls. The Student’s *t-*test at *p* ≤ 0.05 was used to compare the gene expression fold changes using Microsoft^®^ Excel version 15.41. The gene specific primers of all targets were designed on the National Centre for Biotechnology Information (NCBI) database using the Primer-BLAST software [50] and are listed in Supplementary Table S1. These include the sorghum HSP90 (Sb07g028270) [51] and HSP70 (Sb03g039360), a sorghum homologue of an Arabidopsis HSP70 [52,53], the two reference control genes together with ten iTRAQ identified heat stress responsive secreted proteins of ICSB338 sorghum cell suspension cultures.

## 3. Results

### 3.1. Sorghum Cell Suspension Cultures Are Relatively Thermostable at 40 °C

**Figure 1:**
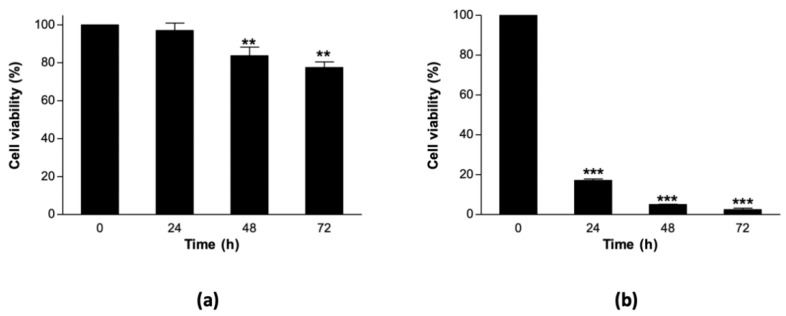
Effects of heat stress on the viability of cell suspension cultures. Cell viability of ICSB338 sorghum (**a**) and Arabidopsis (**b**) cell suspension cultures exposed to heat stress (40 °C) for 72 h. Control cell cultures were maintained at 27 °C and 22 °C for sorghum and Arabidopsis, respectively, and harvested at 0 h. Samples were harvested at 24, 48, and 72 h following the onset of heat stress treatment for cell viability assessment using the MTT assay. Bars represent mean ± SE (*n* = 3). Two or three asterisks indicate significant difference between control and treatment means at ** *p* ≤ 0.01 or *** *p* ≤ 0.001, respectively.

We designed an experiment to assess the level of thermostability of ICSB338 sorghum cell suspension cultures prior to analysing changes in secreted protein abundance and gene expression. In this experiment, control sorghum cell suspension cultures were maintained at 27 °C, while heat stress was imposed at 40 °C for 72 h. We then estimated cell viability at 0, 24, 48, and 72 h using an MTT assay. A moderate but significant decrease in viability was observed in the heat-stressed sorghum cell suspension cultures at 48 and 72 h relative to the control at 0 h (Figure 1a). As this decline was modest and observed from 48 h of treatment, we performed a comparative heat stress experiment using a heat-sensitive plant species, *Arabidopsis thaliana*. Arabidopsis control cell suspension cultures were kept at their normal growth temperature of 22 °C, while heat stress was imposed at 40 °C for 72 h. We observed a drastic decline in the viability of Arabidopsis cell suspension cultures as early as 24 h, which continued to decrease throughout the 72-h heat-stress treatment period (Figure 1b). Overall, these results show that ICSB338 sorghum cell suspension cultures are more thermostable at 40 °C for 72 h when compared to the Arabidopsis cultures.

### 3.2. Heat Stress Upregulates Expression of Sorghum HSP70 and HSP90 Genes

**Figure 2:**
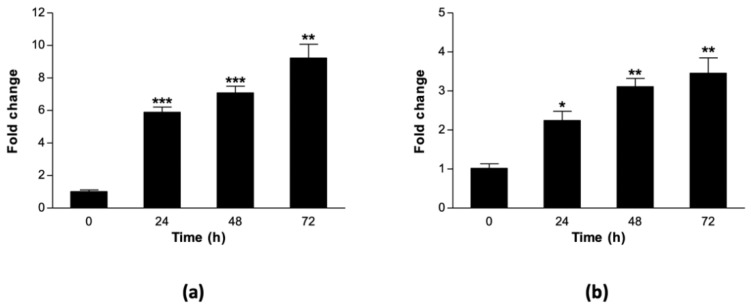
Heat-induced gene expression of sorghum HSP marker genes. ICSB338 sorghum cell suspension cultures growing at mid-log phase were exposed to heat stress at 40 °C for 72 h. Control samples were kept at 27 °C. Cell culture aliquots were samples at 0, 24, 48, and 72 h after heat stress for gene expression analysis using qRT-PCR. Gene expression profile of (**a**) *HSP70* and (**b**) *HSP90.* Bars represent mean ±SE (*n* = 4). One, two, or three asterisks indicate significant difference between control and treatment means at * p ≤ 0.05, ** *p* ≤ 0.01 or *** *p* ≤ 0.001, respectively.

Next, we profiled the heat stress-induced expression of two heat stress marker genes, a sorghum *HSP70 (Sb03g039360)* and *HSP90 (Sb07g028270)*. This experiment was conducted in order to confirm the expected heat stress response of the cell cultures and to establish an appropriate time-point for protein extraction. Sorghum cell suspension cultures were harvested at 0 h, for use as controls, and at 24, 48, and 72 h after imposing heat stress. Four biological replicates were prepared for total RNA extraction, cDNA synthesis and qRT-PCR analysis. There was a linear increase in the expression of both *HSP* genes with time during the heat stress, starting from 24 h and peaking at 72 h (Figure 2). The expression levels of *HSP70* were at least two-fold higher than that of *HSP90* across all time-points (Figure 2a,b). Overall, these results confirmed that the heat stress treatment was sufficient to induce changes in marker gene expression as early as 24 h. Therefore, the 72-h time point was selected for harvesting sorghum cell suspension cultures for secreted protein extraction and analysis.

### 3.3. Heat Stress Triggers Differential Protein Accumulation in the Sorghum Extracellular Matrix (ECM)

ICSB338 sorghum cell suspension cultures were subjected to a heat stress treatment of 40 °C for 72 h, while control samples were maintained at 27 °C. After 72 h, four biological replicate cell suspension cultures were harvested for both the control and heat-stressed samples. Secreted proteins were analyzed via iTRAQ and LC/MS-MS. The mass spectrometry dataset was manually filtered, retaining proteins identified on the basis of at least two sequenced peptides in order to increase the confidence in protein identification. This resulted in the positive identification of 265 ICSB338 sorghum secreted proteins (Table S2).

For the quantitation of differentially accumulated proteins in response to heat stress, the abundance of proteins in the heat-treated samples were computed as fold change, relative to the untreated controls. The data was statistically analyzed using a Student’s *t*-test at *p* ≤ 0.05, resulting in the identification of 100 heat stress responsive sorghum secreted proteins (Tables S3 and S4). We further filtered this dataset, only retaining those with a minimum two-fold change in abundance following heat stress, resulting in 31 differentially accumulated proteins (Table 1). Of these 31 proteins, 55% were up-regulated in response to the stress, while the rest were down-regulated, an indication that heat triggered differential protein accumulation in the sorghum extracellular space. The fold changes of the heat responsive proteins ranged from −6.40 for an uncharacterized protein, SORBI_3002G255000 with an unpredicted protein family name, to 2.85 for a putative germin protein, SORBI_3003G427700. We then analyzed the primary sequences of the 31 heat stress responsive proteins for the presence/absence of signal peptides using SignalP data available on the Uniprot database. The results revealed that 84% of the proteins possessed a signal peptide, while the rest did not. The signal peptide data predicted that the majority of the identified heat stress responsive sorghum proteins are targeted to the secretory pathway (endoplasmic reticulum-Golgi apparatus-extracellular matrix). Alternative pathways for protein secretion are known to exist [54,55,56,57], though not fully understood. An application of the Benjamini–Hochberg procedure [43,44] to the 265 identified proteins with a set false discovery rate of 1% resulted in the identification of 26 differentially accumulated proteins in response to the heat stress (Table S5). Of these 26 proteins, 17 had a minimum fold-change of two and are highlighted in Table 1.

**Figure 3:**
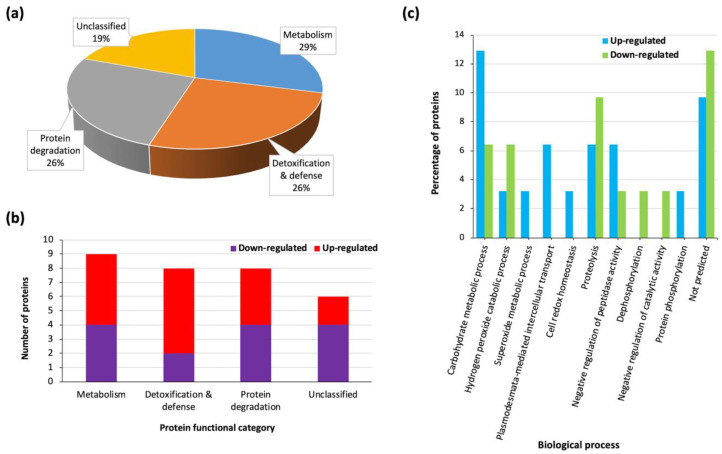
Functional groupings of extracellular sorghum heat stress-responsive proteins. (**a**) Pie chart showing the distribution of proteins into functional categories. (**b**) The number of up- and down-regulated proteins within each protein functional category. (**c**) Distribution of proteins per Gene Ontology Biological process terms.

We also observed that most of the identified heat stress responsive proteins (68%) were uncharacterized (Table 1). Therefore, in order to determine their probable functions during heat response, we further retrieved putative family names or functional domains (where the former where unavailable) of the proteins from the InterPro database as well as Biological process GO terms from the UniProt database and results are shown in Table 1. Examples of the top six enriched families of heat stress responsive proteins include glycosyl hydrolase (5), aspartic peptidase (5), plant peroxidase (3), lipase_GDSL domain (2), germin (2), and leucine-rich repeat domain superfamily (2). The predicted protein families/functional domains were used to group the heat stress responsive proteins into putative functional categories, namely, metabolism (29%), detoxification and defense (26%), and protein degradation (26%), while the rest were unclassified (19%) (Table 1; Figure 3a). The number of up- and down-regulated proteins in each functional group is shown in Figure 3b, while Figure 3c shows the distribution of proteins per GO Biological process. Most proteins involved in carbohydrate metabolic processes were up-regulated (Figure 3c). Overall, these results indicate that ICSB338 sorghum cell suspension cultures responded to heat stress by modulating the abundance of secreted proteins involved in detoxification and defense, metabolism, and protein degradation. However, the biological processes of a large number of the heat stress responsive proteins are yet to be predicted.

### 3.4. Heat Induced Gene Expression Patterns in Sorghum Cell Suspension Cultures

**Figure 4:**
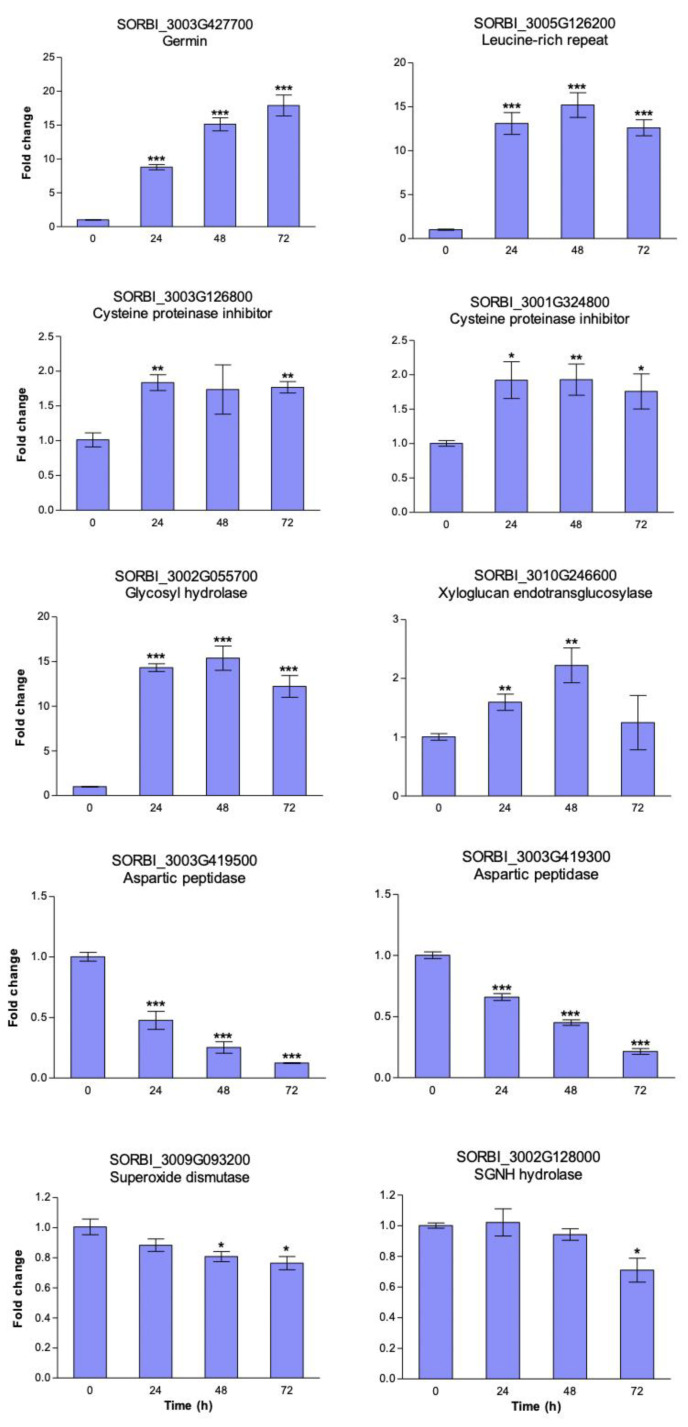
Gene expression analysis of heat-responsive target sorghum genes. ICSB338 sorghum cell suspension cultures were exposed to heat stress at 40 °C for 72 h. Control samples were kept at 27 °C. Cell culture aliquots were sampled at the indicated time-points for RNA extraction and analysis using qRT-PCR. Bars represent mean ± SE (*n* = 4). One, two, or three asterisks indicate significant difference between control and treatment means at * *p* ≤ 0.05, ** *p* ≤ 0.01, or *** *p* ≤ 0.001, respectively.

Next, we validated a subset of iTRAQ identified heat stress-responsive proteins using qRT-PCR analysis. A total of ten genes were selected from the topmost up-regulated proteins for primer design (Table S1). Sorghum cell suspension cultures were exposed to heat stress at 40 °C and cell aliquots were sampled at 0, 24, 48, and 72 h for gene expression analysis. We observed that all the ten target genes responded to the heat stress treatment in at least one time point relative to the controls at 0 h (Figure 4). The genes were either up- or down-regulated during the 72-h heat stress treatment. For example, the *germin* gene (*SORBI_3003g427700*) had a nine-fold increase in expression 24 h after heat stress, which continued to rise, ultimately reaching an eighteen-fold peak at 72 h. Other genes such as the *leucine-rich repeat* (*SORBI_3005g126200*), *cysteine proteinase inhibitors* (*SORBI_3003G126800* and *SORBI_3001G324800*) and a *glycosyl hydrolase* (*SORBI_3002G055700*) were also up-regulated in response to the heat stress treatment. Conversely, the expression of two *aspartic peptidase* genes (*SORBI_3003G419500* and *SORBI_3003G419300*) was suppressed as early at 24 h, reaching a dip at 72 h; while that of a *superoxide dismutase* (*SORBI_3009G093200*) and an *SGNH hydrolase* (*SORBI_3002G128000)* remained unchanged 24 h into the stress treatment followed by a significant decline later during the stress treatment. Overall, these gene expression results indicate that the ten target genes responded to heat at the transcriptional level, validating the iTRAQ data (Table 1).

## 4. Discussion

Heat stress is detrimental to plant growth and productivity, yet climate models are predicting the occurrence of increasingly warmer environmental temperatures [8]. Consequently, research interest in plant stress biology has increased, with studies working towards the identification of genes, proteins, and metabolites for improved thermotolerance in crops [24]. Despite these efforts, our knowledge on contributions of the plant secretome to heat stress response is limited [58], yet such proteins participate in plant growth processes, cell signaling, and in response to unfavorable conditions [33,34,35]. Additionally, the plant apoplast is regarded as a functional space in which extracellular signaling systems are integrated and coordinated prior to responses being elicited [59].

In our study, we focused on heat-induced changes in the secretome of sorghum cell suspension cultures. Since sorghum naturally thrives in hot tropical climates [25,26], it serves as a good model system for studies on heat stress response [31,60]. Previous research has demonstrated the utility of cell cultures as simple and reproducible model systems in secretome studies in response to nutritional deficiency [61], salicylic acid [42,62], and osmotic stress [38]. Others have used cell suspension cultures of pear (*Pyrus communis*) [63], sugarcane (*Saccharum officinarum*) [64], tobacco (*Nicotiana tabacum*) [65], sunflower (*Helianthus annuus*) [58], and rice (*Oryza sativa*) [66] in heat stress research. Of these, only Gammulla et al. [66] investigated the intracellular protein changes of rice cell cultures using LC/MS-MS, while Mita et al. [58] studied both intra- and extra-cellular protein changes of sunflower cultured cells using ^35^S-methionine protein labelling, gel-based protein separation methods and Western blotting, without the MS-based identification of target proteins. Therefore, there is a need to improve our understanding of plant secretome functions during heat stress response.

Estimates of cell growth, membrane integrity and/or metabolic activity of cell suspension cultures during heat treatment are proxies of cell vitality [63]. We observed relatively high and sustained metabolic activity in ICSB338 sorghum cell suspension cultures following 72 h of heat treatment (40 °C), while that of Arabidopsis cultures significantly dropped under similar conditions (Figure 1). These results indicate that sorghum cell suspension cultures retained enzyme activity at 40 °C for 72 h, while the same treatment was lethal to Arabidopsis cultures. We then used ICSB338 sorghum cell suspension cultures to investigate changes in gene expression and secreted protein abundance in response to heat stress.

Apart from altering metabolic processes (Figure 1), heat stress also induced changes in gene expression (Figure 2). Heat stress marker genes, *HSP70* and *HSP90*, were up-regulated in sorghum cell suspension cultures exposed to 40 °C for 72 h relative to the controls at 27 °C (Figure 2). HSPs are well-characterised stress proteins that protect cell macromolecules against heat damage [16]. When plants are exposed to elevated temperatures, they reprogram their gene expression for survival and maintenance of cellular homeostasis [23]. Products of such transcriptional changes include signaling (kinases, phosphatases, and phospholipases) and protective (HSPs, antioxidant enzymes) proteins [9,18,23,67], as well as osmoregulatory metabolites [68]. We used activation of HSP gene expression as a marker to confirm that the cells had sensed the heat stress and activated an appropriate molecular response. Due to different kinetic profiles of transcript and protein accumulation, it is quite possible that these HSPs might peak at a different time point than their transcripts. However, because we were not focusing on any particular response pathway, we selected 72 h on the basis that the cells had sensed and responded to the stress. The observed up-regulation of *HSP70* and *HSP90* genes in our study correlates well with known transcriptional changes in response to heat stress and provides evidence that the imposed heat stress treatment modulates molecular processes.

Plant cells also secrete proteins into the ECM in response to changing environments [38,42,61,62]. However, there are two critical factors that should be considered when reviewing quantitative biological data. Modest changes in gene expression and/or protein accumulation may have significant biological significance, while massive activation of gene expression may represent consequences rather than a cause for phenomena under investigation. Thus, we applied a two-fold cut-off threshold to filter our data (Table 1), but we have provided the entire dataset in Tables S3 and S4. Only follow-up genetic experiments will validate biologically relevant proteins rather than artificial thresholds based on magnitude of change. We identified 31 secreted proteins that differentially accumulated in the sorghum ECM in response to heat stress with a minimum two-fold change in abundance (Table 1). Of these, 84% possessed predicted signal peptides, which are vital for the classical secretory pathway via the endoplasmic reticulum-Golgi apparatus-extracellular matrix route. Examples of such proteins include several members of glycosyl/glucoside hydrolase, peptidase and protease inhibitor families, class III peroxidases, germin, GDSL/SGNH lipases, and leucine-rich repeat domain containing proteins (Table 1). A minority of the heat stress responsive proteins including Mn/Fe superoxide dismutase and purple acid phosphatase lacked predicted signal peptides (Table 1) and could possibly be classified as leaderless proteins [35,54,56,57,69]. Signal peptide-lacking purple acid phosphatases have previously been identified in a secretome analysis of white sorghum cell suspension cultures responding to osmotic stress [38], and a broader list of putative leaderless proteins of plant origin is published elsewhere [54]. As discussed by Ding et al. [54], the identification of such proteins in independent plant secretome studies highlights the possible existence of non-classical secretory pathways and warrants further investigation.

Secretome studies using experiments employing stress treatments are dogged with concerns relating to stress-induced death of some cells and appearance of non-biologically relevant proteins in the extracellular matrix. While our study raises the same concerns, partly due to the observed reduction in cell viability at 72 h relative to the untreated controls and the first 24 h of heat treatment (Figure 1a), there are two critical points in mitigation. Firstly, all the proteins increasing in abundance were not newly synthesized and did not appear only in the heat-stressed samples but were already present in the untreated controls. This makes the possibility of damage-induced leakage of proteins, as a result of reduced cell vitality at 72 h and their subsequent identification as differentially accumulated proteins, an improbable proposition. Secondly, we did not identify any major cytosolic proteins not previously associated with the extracellular matrix in other studies using cell cultures without stress. We also provided information on the presence/absence of a secretory signal peptide showing strong probability that a majority of these proteins identified at 72 h post heat stress treatment are secreted via the classical pathway. However, proteomic studies of this nature should, wherever possible, analyze changes in protein accumulation at multiple time-points in order to compare temporal variations in stress response.

The identified heat responsive sorghum secreted proteins have putative functions in metabolism (29%), detoxification and defense (26%), and protein degradation (26%), while 19% were unclassified (Figure 3a). In total, 75% of the detoxification and defense-related proteins were up-regulated in response to heat stress (Figure 3b). These proteins include germin and superoxide dismutase (Table 1), which produce H_2_0_2_ in independent reactions [70,71,72], as well as class III peroxidases that detoxify H_2_0_2_ in downstream processes [73,74]. While lower levels of H_2_0_2_ may function as signaling molecules during stress response, its increased accumulation and that of other reactive oxygen species (ROS) results in oxidative stress, which is detrimental to cell structure and function [75,76]. Our study indicates that heat stress triggers the secretion of antioxidant enzymes into the ECM, possibly to quench increased ROS levels at elevated temperatures. Such oxidative bursts have been implicated in plant-pathogen interaction [77]. Similarly, numerous redox-related proteins were up-regulated in the sorghum secretome following osmotic stress [38], thus indicating common response mechanisms towards different stresses [13].

The metabolism category was dominated by six carbohydrate hydrolyzing enzymes and three lipases (Table 1). While the majority (4) of the glycosyl/glycoside hydrolases were up-regulated, two lipase-GDSL domain proteins were down-regulated. Carbohydrates are structurally and functionally diverse molecules, and their hydrolysis is catalyzed by a wide spectrum of enzymes during cell wall modification and turnover of signaling molecules [78], amongst others. The significance of the identified carbohydrate metabolizing enzymes in heat stress response is still unclear and requires further analyses using metabolomics and gene functional studies. However, glycosyl/glycoside hydrolases were similarly up-regulated in a sorghum secretome study in response to osmotic stress [38], thus underscoring their importance in ECM responses to abiotic stresses.

Lipids are important structural components of biological membranes and participate in signaling processes in response to biotic and abiotic stresses [79]. Three GDSL/SGNH domain containing lipases, which hydrolyze diverse substrates including sulphur, amino acids, and lipids [80] were identified in this study. Of these lipases, an SGNH lipase (SORBI_3002G128000) was up-regulated in our study, while two GDSL lipases were down-regulated (Table 1). The same SGNH lipase protein was also up-regulated in a sorghum secretome in response to osmotic stress, while the other 2 GDSL lipases were not identified [81]. The up-regulation of the SGNH lipase in response to both stresses possibly highlights basal mechanisms for plant survival during unfavorable conditions. In another secretome analysis of Arabidopsis cell suspension cultures in response to salicylic acid, a GDSL lipase named GLIP1 was up-regulated and later shown to protect plants against the fungus *Alternaria brassicicola* [62]. Due to the broad substrate specificity of GDSL/SGNH lipases in plants [80], more functional studies are required in order to elucidate their specific roles during heat stress.

The third functional group identified in our study consisted of protein degradation-related proteins (Figure 3a), with five peptidases and three protease inhibitors (Table 1). The plant ECM in known to contain proteolytic enzymes [35,82] and the rate of protein degradation in biological systems is regulated by activities of proteases and their inhibitors [83]. Protein degradation plays a role in environmental stress response, including heat, by removing abnormally folded/misfolded/aggregated proteins and those damaged by oxidative stress [83,84]. The identified proteases and their inhibitors could possibly function in regulating the removal of heat/oxidative stress-damaged proteins. Additionally, proteolysis is an important regulatory mechanism to activate peptide signals [85]. For example, small post-translationally modified peptides require proteolytic processing from precursor proteins for the generation of mature active forms [86,87]. Overall, the presence of heat-responsive peptidases and protease inhibitors in our study highlights the need for regulating protein turnover and/or activity during heat stress response.

We validated ten iTRAQ identified heat stress-responsive proteins using qRT-PCR in a time-course experiment (Figure 4). All target genes were differentially expressed at some point, indicating their transcriptional regulation during heat stress. Of particular note is the remarkable activation of the germin gene (Figure 4), which correlates well with the iTRAQ results (Table 1). We also made a curious observation that gene expression for aspartic peptidase proteins with Accession numbers C5XHP9 and C5XHP7 was down-regulated in response to heat stress (Figure 4), while the proteins were up-regulated by heat (Table 1). A similar lack of correlation between gene expression and protein accumulation has been previously reported [88,89] with a number of possible scenarios to account for this being proposed [90]. Because protein stability and turnover are controlled by different cellular machineries, suppression of a critical component responsible for degradation of specific proteins could lead to an apparent increase in protein abundance (when it is increase stability) in the face of a real transcriptional suppression. The reverse is also true, when activation of gene expression does not result in increased protein abundance due to increased activity in degradation of specific polypeptides. This underlines why it is important to validate transcriptomic data with protein measurements wherever possible.

We have provided the entire protein dataset filtered by *p-*value (Tables S3 and S4) and an arbitrary cut-off threshold (Table 1). Because of the nature of the statistical analysis and the significance level applied, this means that 5% of the differentially accumulated heat stress responsive candidates would be false positives. However, for future reverse genetic experiments, application of a stringent Benjamini–Hochberg false discovery rate of 1% filters the candidate list down to 26 target proteins (Table 1, Table S5). This would be a practical way to select candidates for further analysis from such a large dataset [44]. Overall, the different expression profiles observed in our study highlight individual gene and/or gene family-specific responses to heat stress. However, further functional studies are required in order elucidate specific roles of these targets in heat stress adaptive mechanisms.

## 5. Conclusions

Although intracellular proteomic studies have broadened our understanding on plant processes involved in heat stress response, equivalent information on the plant secretome in currently unavailable. Our study provides some insight into possible functions of sorghum secreted proteins in ROS detoxification, carbohydrate, and lipid metabolism, as well as proteolysis under high temperature stress. The results also support the notion that elevated temperatures increase the production of ROS, whose destructive effects are minimized by various antioxidant enzymes. Carbohydrates and lipids, as integral components of cell walls and membranes, are also metabolized during heat stress response, possibly during cell wall modification and/or recycling of signaling molecules. Additionally, the ECM is site for tightly regulated and selective protein degradation processes facilitated by proteases and their inhibitors. We acknowledge the need for functional studies to ascertain the roles of these proteins/genes in heat stress response. Nonetheless, our study suggests that the sorghum ECM is a “gold mine” for novel heat stress markers such as germin (SORBI_3003G427700), which is highly heat-inducible at both protein and gene level. We also acknowledge that the differentially accumulated proteins of this study could be a combination of changes reflecting the consequences of heat stress, due to the observed reduction in cell viability at 72 h and the protein changes enabling cells to survive heat. Therefore, proteomic analyses at multiple time-points during the stress treatment period are recommended. Furthermore, the Benjamini–Hochberg procedure could be applied in order to filter such large datasets prior to further analysis. Nevertheless, our study has provided data for other researchers in the field to select candidate proteins for further functional validation studies. Some of the identified sorghum target proteins/genes could be important in the genetic improvement of crops for enhanced heat tolerance.

## Figures and Tables

**Table 1:** List of differentially accumulated sorghum secreted proteins in response to heat stress.

Prot No. ^a^	Accession ^b^	Protein Name	Ratio ^c^	SD ^d^	*p*-Value ^e^	Signal Peptide ^f^	Family Name ^g^
**Metabolism**							
11	A0A1B6QCB0 *	Alpha-amylase OS = *Sorghum bicolor* GN = SORBI_3002G190500	2.84	0.22	4.89 × 10^−5^	+	Alpha-amylase, plant
36	C5XB39 *	Uncharacterized protein OS = *Sorghum bicolor* GN = SORBI_3002G055700	2.41	0.20	3.80 × 10^−4^	+	Glycosyl hydrolase superfamily
92	C5XD22	Uncharacterized protein OS = *Sorghum bicolor* GN = SORBI_3002G356800	−2.32	0.05	2.54 × 10^−3^	+	Lipase_GDSL domain
106	A0A1W0VY92 *	Uncharacterized protein OS = *Sorghum bicolor* GN = SORBI_3003G205900	−2.31	0.08	4.37 × 10^−4^	+	Lipase_GDSL domain
113	A0A1B6Q537	Uncharacterized protein OS = *Sorghum bicolor* GN = SORBI_3003G244600	2.02	0.47	5.86 × 10^−3^	-	Glycosyl hydrolase, family 18
125	C5WSY5	Uncharacterized protein OS = *Sorghum bicolor* GN = SORBI_3001G014700	−2.51	0.07	4.89 × 10^−3^	+	Glycoside hydrolase, family 17
127	C5Z8T4 *	Xyloglucan endotransglucosylase/hydrolase OS = *Sorghum bicolor* GN = SORBI_3010G246600	2.47	0.30	4.69 × 10^−4^	+	Xyloglucan endotransglucosylase/hydrolase
250	C5 × 578	Uncharacterized protein OS = *Sorghum bicolor* GN = SORBI_3002G128000	2.31	0.33	1.32 × 10^−2^	+	SGNH hydrolase superfamily
279	A0A1B6QEM0	Xyloglucan endotransglucosylase/hydrolase OS = *Sorghum bicolor* GN = SORBI_3002G324100	−2.50	0.10	2.61 ×10 ^−2^	+	Xyloglucan endotransglucosylase/hydrolase
**Detoxification & Defence**							
15	C5X5K6	Peroxidase OS = *Sorghum bicolor* GN = SORBI_3002G416700	−2.17	0.02	1.34 × 10^−2^	+	Plant peroxidase
50	C5X040 *	Peroxidase OS = *Sorghum bicolor* GN = SORBI_3001G080300	2.06	0.27	5.18 × 10^−4^	+	Plant peroxidase
58	C5YVR0 *	Superoxide dismutase OS = *Sorghum bicolor* GN = SORBI_3009G093200	2.35	0.38	8.83 × 10^−4^	-	Manganese/iron superoxide dismutase
60	C5XHF1 *	Uncharacterized protein OS = *Sorghum bicolor* GN = SORBI_3003G136200	2.08	0.24	2.38 × 10^−4^	+	Germin
108	C5XL59 *	Peroxidase OS = *Sorghum bicolor* GN = SORBI_3003G024700	−3.23	0.07	2.70 × 10^−5^	-	Plant peroxidase
137	C5XHX2 *	Uncharacterized protein OS = *Sorghum bicolor* GN = SORBI_3003G427700	2.85	0.43	4.85 × 10^−4^	+	Germin
154	C5XJT8 *	Uncharacterized protein OS = *Sorghum bicolor* GN = SORBI_3003G156400	2.17	0.29	4.45 × 10^−4^	+	Thioredoxin-like superfamily
166	C5YD83	Uncharacterized protein OS = *Sorghum bicolor* GN = SORBI_3006G031200	2.09	0.45	7.25 × 10^−3^	+	Thioredoxin-like superfamily
**Protein Degradation**							
47	C5XHP9 *	Uncharacterized protein OS = *Sorghum bicolor* GN = SORBI_3003G419500	2.18	0.13	5.94× 10^−5^	+	Aspartic peptidase A1 family
49	C5XHP7 *	Uncharacterized protein OS = *Sorghum bicolor* GN = SORBI_3003G419300	2.74	0.20	3.77 × 10^−5^	-	Aspartic peptidase A1 family
74	C5WVG9 *	Cysteine proteinase inhibitor OS = *Sorghum bicolor* GN = SORBI_3001G324800	2.19	0.17	8.33 × 10^−5^	+	Cysteine proteinase inhibitor
153	C5Z1X3	Uncharacterized protein OS = *Sorghum bicolor* GN = SORBI_3010G268400	−2.10	0.03	5.84 × 10^−3^	+	Aspartic peptidase A1 family
215	C5XAQ7	Uncharacterized protein OS = *Sorghum bicolor* GN = SORBI_3002G326100	−2.05	0.08	4.50 × 10^−2^	+	Aspartic peptidase A1 family
219	A0A1B6Q242	Uncharacterized protein OS = *Sorghum bicolor* GN = SORBI_3003G085300	−2.00	0.03	5.30 × 10^−3^	+	Bowman-Birk type proteinase inhibitor
225	C5XGM0	Cysteine proteinase inhibitor OS = *Sorghum* *bicolor* GN = SORBI_3003G126800	2.16	0.42	9.39 × 10^−3^	+	Cysteine proteinase inhibitor
259	A0A1B6Q6G6	Uncharacterized protein OS = *Sorghum bicolor* GN = SORBI_3003G314800	−2.00	0.11	2.50 × 10^−2^	+	Aspartic peptidase A1 family
**Unclassified**							
26	A0A1Z5R915 *	Purple acid phosphatase OS = *Sorghum bicolor* GN = SORBI_3007G091100	−4.18	0.04	2.42 × 10^−4^	-	Purple acid phosphatase-like
33	C5XBP7	Uncharacterized protein OS = *Sorghum bicolor* GN = SORBI_3002G343600	−2.55	0.06	3.77 × 10^−3^	+	Leucine-rich repeat domain superfamily
34	A0A1B6PLT5 *	Uncharacterized protein OS = *Sorghum bicolor* GN = SORBI_3006G133000	−2.83	0.05	5.29 × 10^−4^	+	Domain of unknown function DUF642
101	C5Y2R8 *	Uncharacterized protein OS = *Sorghum bicolor* GN = SORBI_3005G126200	2.48	0.37	6.26 × 10^−4^	+	Leucine-rich repeat domain superfamily
268	C5X4M5	Uncharacterized protein OS = *Sorghum bicolor* GN = SORBI_3002G255000	−6.40	0.10	8.97 × 10^−3^	+	Not predicted
273	C5WPH2 *	Uncharacterized protein OS = *Sorghum bicolor* GN = SORBI_3001G130400	2.34	0.35	6.69 × 10^−4^	+	Protein of unknown function DUF538

^a^ Protein number assigned in ProteinPilot software. ^b^ Protein accession numbers obtained from the UniProt database searches against sequences of *Sorghum bicolor* only. ^c^ Ratio represents the average fold-change (*n* = 4) in response to heat stress (40 °C) relative to the control (27 °C). A negative value indicates down-regulation. Only proteins with a minimum two-fold change in abundance were retained. ^d^ Standard deviation of the fold-changes (*n* = 4). ^e^ Probability value obtained from a Student’s *t*-test comparing the fold changes between the control and heat stress treatment means (*n* = 4). ^f^ Signal peptide predicted using SignalP 4.1 data available on the Uniprot database; + denotes the presence of a signal peptide in the primary sequence of the protein, while – denotes the absence of a signal peptide. ^g^ Family name (or functional domains) as predicted using the InterPro (http://www.ebi.ac.uk/interpro/). * Proteins with a change in abundance in response to heat stress according to the Benjamini–Hochberg procedure using a false discovery rate of 1%. These proteins appear amongst the list of 31 heat stress responsive proteins with a minimum two-fold change in abundance.

## Supplementary Materials

The following are available online at https://www.mdpi.com/2227-7382/8/4/29/s1, Table S1: List of sorghum primer sequences used in gene expression analysis, Table S2: Details of proteins identified in the extracellular matrix of ICSB338 sorghum cell suspension cultures, Table S3: Details of the 100-heat stress responsive ICSB338 sorghum secreted proteins, Table S4: List of all 100 differentially accumulated sorghum secreted proteins in response to heat stress, Table S5: The Benjamini–Hochberg critical values of the 265 identified sorghum secreted proteins.
