# Supplementary material for: Heat Stress Triggers Differential Protein Accumulation in the Extracellular Matrix of Sorghum Cell Suspension Cultures
Source: Proteomes. 2020 Oct 22;8(4):29. doi: 10.3390/proteomes8040029 (PMC7709130; doi:10.3390/proteomes8040029)
Supplement: Supplementary file 1 [file proteomes-08-00029-s001.zip › Table S1.docx]

**Table S1:** List of sorghum primer sequences used in gene expression analysis.

| **Accession no.** | **Gene ID** | **Protein family name** | **Forward primer (5'>3')** | **Reverse Primer (5'>3')** |
| --- | --- | --- | --- | --- |
| C5XHX2 | SORBI_3003G427700 | Germin | GAGCTTCCAGATCGACGGAG | TGAACATGAACATGGCAGGC |
| C5Y2R8 | SORBI_3005G126200 | Leucine-rich repeat | CGGTTCCATCGGAAGTCCTC | CATGCAGTCTTCAGCGCATC |
| C5XGM0 | SORBI_3003G126800 | Cysteine proteinase inhibitor | TTGCTTGCGAGCAGTGTATG | TGGAGCTCGCATGTTCACTC |
| C5WVG9 | SORBI_3001G324800 | Cysteine proteinase inhibitor | ACTCCTCCCTGCCTCTATGG | CAGCGACTTGCAGTGAAACC |
| C5XB39 | SORBI_3002G055700 | Glycosyl hydrolase | GAGGATCACACCGAGACCAC | GTTGTTTTTGGCTGTCCGGC |
| C5Z8T4 | SORBI_3010G246600 | Xyloglucan endotransglucosylase | GGCACTTCAATTCCTCGCAC | TTCGTTCGTTTTGTTCGGGC |
| C5XHP9 | SORBI_3003G419500 | Aspartic peptidase | TCAACAGAGCTCGGTGAAATC | TGCATGCGTCCAAAAATTCG |
| C5XHP7 | SORBI_3003G419300 | Aspartic peptidase | GGTGATCATCGGAGGGTTCC | TCGGTGTACCCTACCTAGCC |
| C5YVR0 | SORBI_3009G093200 | Superoxide dismutase | GAAACTTGGCTGGGCCATTG | CAGCGCCTTCTGCATTCATC |
| C5X578 | SORBI_3002G128000 | SGNH hydrolase | TGCTATTCAAACCGCCATCG | TAAGGTTGCGACACTTTTGC |
| HSP70 | Sb03g039360 | Heat shock protein 70 | TCATATCGTTGCCTTTCGTGTTG | CACTTGATTCTCTTCGTACAGTTTG |
| HSP90 | Sb07g028270 | Heat shock protein 90 | GCTGGATGCGTGTGTTATCG | TGAAAGACAGCAGGATAAACGG |
| *Ref. Gene | Sb03g038910 | Uncharacterised protein | TCCTGAAGCATCTTTCCCTCC | ACAGCCTGATTAGTTGGGGG |
| *Ref. Gene | Sb04g003390 | Eukaryotic initiation factor-4A | GATGAGATGCTCTCCCGTGG | TGATCTCTAGGGCCTCTGGG |

* Reference control genes
